# Anisotropic magnetic entropy change in *R*FeO_3_ single crystals(*R* = Tb, Tm, or Y)

**DOI:** 10.1038/srep19775

**Published:** 2016-01-25

**Authors:** Ya-Jiao Ke, Xiang-Qun Zhang, Yue Ma, Zhao-Hua Cheng

**Affiliations:** 1 State Key Laboratory of Magnetism and Beijing National Laboratory for Condensed Matter Physics, Institute of Physics, Chinese Academy of Sciences, Beijing 100190, China

## Abstract

**Compared with traditional gas-compression/expansion refrigeration, magnetic refrigeration based on magnetocaloric effect (MCE) exhibits the advantages of high energy efficiency and environment friendliness. Here, we created large MCE in RFeO**_**3**_ **(R = Tb or Tm) single crystals by the magnetization vector rotation of single crystal with strong magnetocrystalline anisotropy (MCA), rather than merely via the order-disorder magnetic phase transition or magnetic structural transition. Owing to the difference in charge distribution of 4*****f*****-electrons between Tb**^**3+ **^** and Tm**^**3**+^ **ions, the rotating field entropy with different signs,** −Δ***S***_***M***_^***R***^** = 17.42 J/kg K, and** –Δ***S***_***M***_^***R***^** = −9.01 J/kg K are achieved at 9 K and 17 K for TbFeO**_**3 **_**and TmFeO**_**3**_ **single crystals from** ***b*** **axis to** ***c*** **axis, at 50 kOe, respectively. The finding of the large anisotropic MCE not only advances our understanding of the anisotropy of MCE, but also extends the application for single crystals to magnetic refrigeration.**

Magnetocaloric effect (MCE), which describes the temperature change of magnetic materials in an adiabatic process caused by magnetic entropy change Δ*S*_*M*_ under external magnetic field, has been extensively investigated. In comparison with traditional gas-compression/expansion refrigeration, magnetic refrigeration based on MCE exhibits the advantages of high energy efficiency and environment friendliness. The giant or very large magnetic entropy change was obtained in various kinds of magnetic materials, including Gd-based alloys Gd_5_(Si_*x*_Ge_1-*x*)_^1^,^2^, Mn-based Ni-Mn-Ga(Sn) alloys^3^,^4^ and MnFeP_0.45_As_0.55_^5^, Fe-based LaFe_13-*x*_(Si, Al)_*x*_^6^,^7^, as well as rare-earth perovskite-type manganites (La_1-*x*_M_*x*_)MnO_3_ (M = Ca, Sr, and Ba etc.)^8^,^9^. Although numerous studies on MCE have been concentrated on exploring new materials with giant MCE near room temperature for domestic applications, giant MCE in the low-temperature region from about 30 K down to sub-Kelvin temperatures is also essential for utilization in certain fields, such as liquid hydrogen economy and space application^10^.

The magnetic, barocaloric and electrocaloric effects can be tuned or created by element substitution^11^, pressure^12^,^13^,^14^,^15^, strain^16^,^17^, electric field^18^,^19^, or elastic force^20^. The giant magnetic entropy change in the vicinity of magnetic ordering temperature is usually accompanied by a field-induced or temperature-induced magnetic phase transition with the changes in either crystal symmetry or volume^21^. In addition to magnetic entropy change, mechanical properties and chemical stability are key issues for the practical use of magnetic refrigerator^22^. The material will definitely become very brittle and even break into smaller grains if its crystal symmetry or volume is changed very frequently, and consequently the corrosion resistance and the lifetime of a magnetic refrigerator will be deteriorated. Therefore, it is interesting to explore whether the giant MCE can be created by the magnetization vector rotation of single crystal with strong magnetocrystalline anisotropy (MCA), rather than merely via the order-disorder magnetic phase transition or magnetic structural transition.

Although the anisotropic MCE, which was discovered in Ni single crystal more than 70 years ago^23^, is lower than that from the paramagnetic-ferromagnetic phase transition, it should be large for materials with high values of derivatives of the MCA with respect to temperature^24^,^25^,^26^,^27^,^28^,^29^,^30^,^31^,^32^,^33^,^34^,^35^. Here, we explore the anisotropic magnetic entropy change of *R*FeO_3_ single crystals with *R* = Tb, Tm or Y. The reasons for choosing *R*FeO_3_(*R* = Tb, Tm, or Y) single crystals are three-fold. Firstly, *R*FeO_3_ show a complex magnetic transformation and spin-reorientation transitions^36^. The magnetoelectric properties and superfast optomagnetic effect of *R*FeO_3_ single crystal have been extensively investigated^37^,^38^. Unfortunately, the effect of the complex magnetic transformation on MCE is not understood yet. Secondly, the magnetic moments of Tb^3+^ ion and Tm^3+^ ion are large, and we can achieve a larger magnetic entropy change in *R*FeO_3_(*R* = Tb, Tm) single crystal according to the relation of *−*Δ*S*_*M*_^*Max*^ = Rln(2*J* + 1), where *R* is the gas constant and *J* is the total angular momentum of the magnetic ion. Thirdly, the 4*f* shell of Tb^3+^, Tm^3+^ and Y^3+^ has an oblate, a prolate, and a spherical shape, respectively, a different anisotropy of MCE would be expected between the TbFeO_3_ and TmFeO_3_ single crystals on the basis of single-ion-anisotropy model^39^. The rotating field entropy with different signs, *−*Δ*S*_*M*_^*R*^ = 17.42 J/kg K, and *−*Δ*S*_*M*_^*R*^ = −9.01 J/kg K are achieved at 9 K and 17 K for TbFeO_3_ and TmFeO_3_ single crystals from *b* axis to *c* axis, respectively. The finding not only advances our understanding of the MCE anisotropy in magnetic single crystals, but also opens a new arena for magnetic refrigerator by rotating its magnetization vector.

X-ray diffraction (XRD) patterns and back-reflection Laue XRD patterns demonstrate that RFeO_3_(R = Tb, Tm or Y) single crystals have an orthorhombically distorted pervoskite structure with *P*bnm symmetry (not shown). Figure 1(a,b) display the zero-field-cooled (ZFC) and field-cooled (FC) thermal magnetization curves along *a*, *c* axes from 2 K to 300 K under a magnetic field of 100 Oe for TbFeO_3 _and TmFeO_3_ single crystals, respectively. The kink point at 3 K indicated by the arrows in inset of Fig. 1(a) corresponds to the ordering temperature of Tb^3+^ moments (*T*_*N*_^*Tb*^). From the inset thermal magnetization curves of *a* and *c* axes, two spin-reorientation transitions are observed in the temperature range from 8.5 K to 6 K and 3.5 K to 2.5 K, corresponding to the spin-reorientation of Fe^3+^ moments from Γ_4_(*G*_*x*_,*A*_*y*_,*F*_*z*_) configuration to Γ_2_(*F*_*x*_,*C*_*y*_,*G*_*z*_) configuration, and then back to the high temperature configuration Γ_4_(*G*_*x*_,*A*_*y*_,*F*_*z*_)^36^,^37^,^40^. From the thermal magnetization curves of *a* and *c* axes of TmFeO_3_ single crystal shown in Fig. 1(b), a spin-reorientation transitions is observed in the temperature range from 85 K to 95 K, corresponding to the spin-reorientation of Fe^3+^ moments rotate from Γ_4_(*G*_*x*_,*A*_*y*_,*F*_*z*_) configuration to Γ_2_(*F*_*x*_,*C*_*y*_,*G*_*z*_) configuration^36^.

Figure 2(a–c) illustrate the isothermal magnetization curves along *a*, *b* and *c* axes of TbFeO_3_ single crystal in the temperature range of 2–40 K with an interval of 2 K, respectively. The magnetization curves for these three directions are different either in shape or in magnetization values. Data for increasing and decreasing the magnetic field at 2 K to 10 K along all the three directions are given, for better viewing we enlarged the data along b axis in the inset, which demonstrates a little hysteresis loss in the cycling process. A spin-flip phenomenon can be observe along *a*, *b* and *c* axis of the TbFeO_3_ single crystal at 2 K due to the antiferromagnetic interaction of Tb-Tb ions^40^,^41^. Form the data we can see that the easy magnetization direction (EMD) lies in *ab* plane for TbFeO_3_ single crystal. The significant difference in the isothermal magnetization curves along *a*, *b* and *c* axis of TbFeO_3_ single crystal implies that an anisotropic MCE can be expected.

At temperature *T*, the magnetic entropy change due to applied field *H* can be calculated from the isothermal curves by the Maxwell relation


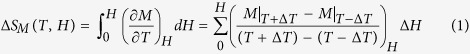


where the slope of two adjacent data points is approximatively used for the numerical calculation of the gradient of *(∂M/∂T)*_*H*_.

By selecting Δ*T* = 1 K and Δ*H* = 2 kOe, the calculated *−*Δ*S*_*M*_ vs temperature is shown in Fig. 2(d–f) for fields along *a*, *b* and *c* axis, respectively. A large anisotropy of MCE is observed in TbFeO_3_ single crystal along *ab* plane and *c* axis. The maximum values of *−*Δ*S*_*M*_ are achieved of 24.05 J/kg K and 20.18 J/kg K in a field of 70 kOe at 11 K along *a* axis and 9 K along *b* axis, respectively. The values of *−*Δ*S*_*M*_ along *c* axis are smaller than those along *a* and *b* axis above the ordering temperature of Tb^3+^ moments (*T*_*N*_^*Tb*^ ~ 3K). Around *T*_*N*_^*Tb*^ ~ 3K, a field-induced transition from antiferromagnetic configuration of Tb^3+^ moments to ferromagnetic one results in *−*Δ*S*_*M*_ = 10.55 J/kg K.

Figure 3(a–c) illustrate the isothermal magnetization curves along *a*, *b* and *c* axis of TmFeO_3_ single crystal in the temperature range of 2–40 K with an interval of 2 K, respectively. In contrast to TbFeO_3_ single crystal, TmFeO_3_ single crystal exhibits a uniaxial magnetic anisotropy with EMD along *c*-axis. The magnetic entropy change *−*Δ*S*_*M*_ calculated from the isothermal curves using the equation (1) is shown in Fig. 3(d–f) for fields along the *a*, *b* and *c* axis, respectively. The maximum values of *−*Δ*S*_*M*_ are achieved of 11.93 J/kg K in a field of 70 kOe at 17 K along *c* axis, whereas *−*Δ*S*_*M*_ for *a* and *b* axes are about one order of magnitude smaller than those along *c* axis in the whole temperature range.

The anisotropy of magnetic entropy change results from the MCA. In general, the overall MCA of *R*FeO_3_ single crystal is the sum of *R*^3+^ sublattice anisotropy and Fe^3+^ sublattice one, as similar with *R*MnO_3_ series^29^. In order to separate the individual contribution from R^3+^ ion sublattice, we measured the magnetization curves and magnetic entropy change of YFeO_3_ single crystal for comparison. Since Y ion has non-magnetic moments, and consequently makes no contribution to the overall MCA. Therefore, it affords a separate investigation of the Fe^3+^ sublattice anisotropy. Isothermal magnetization curves along *a*, *b* and *c* axis of YFeO_3_ single crystal are shown in Fig. 4(a–c), respectively. The magnetization curves indicate that the magnetic anisotropy among *a*, *b* and *c* axis is not significant. Furthermore, the magnetic entropy change of YFeO_3_ are nearly zero (Fig. 4(d–f)), suggesting that the anisotropy of magnetic entropy change in TbFeO_3_ and TmFeO_3_ single crystals is arisen mainly from the contribution of Tb^3+^ and Tm^3+^ ions sublattice anisotropy.

In the first approximation, the MCA constant *K*_1,R_ can be described as^42^.


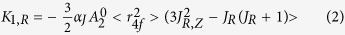


where *α*_*J*_ is the second-order Stevens coefficient, and 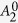 is the second-order crystalline electrical field (CEF) coefficient. 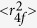 is the squared 4*f* shell radius. *J*_R_ is the Hund’s rules angular moment of R ion.

Since the sign of 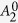 for orthorhombically distorted pervoskite structure RFeO_3_(R = Tb, Tm or Y) single crystals is the same and negative^43^, the easy magnetization directions of these single crystals are governed by the sign of the second-order Stevens factor *α*_*J*_ of rare earth ions. The signs of *α*_*J*_ for Tb^3+^ and Tm^3+^ are negative and positive, respectively. Therefore, the MCA constants *K*_1,Tb_ < 0, and *K*_1,Tm_ > 0, suggesting that the easy magnetization direction of TbFeO_3_ and TmFeO_3_ single crystals aligns in *ab* plane and *c* axis, respectively. Similar results were also observed in DyFeO_3_ and ErFeO_3_ single crystals^33^,^34^.

The connection between anisotropic magnetic entropy change and magnetic anisotropy is evident from the field-dependence of *−*Δ*S*_*M*_ for TbFeO_3_ single crystal at 9 K and TmFeO_3_ single crystal at 17 K along different axis (Fig. 5(a,c)). For magnetic refrigeration application, not only a large entropy change value, but also a large refrigeration capacity (RC) is required. *RC* is defined as


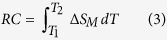


where *T*_1_ and *T*_2_ are the temperatures corresponding to both sides of the half-maximum value of *−*Δ*S*_*M*_ peak. The *RC* is the measure of the amount of heat transfer between the cold and hot reservoirs in an ideal refrigerator as a function of field. The field-dependent refrigeration capacity of TbFeO_3_ and TmFeO_3_ single crystals is shown in Fig. 5(b) and Fig. 5(d). The three directions manifest obvious anisotropy with values of *RC* in a field of 70 kOe are 504.8 J/kg, 319.9 J /kg and 11.4 J/kg for *a*, *b* and *c* axes for TbFeO_3_ single crystal, respectively. For TmFeO_3_ single crystal, we also see obvious anisotropy with values of *RC* in a field of 70 kOe are 34.8 J/kg, 47.8 J/kg and 279.2 J/kg for *a, b* and *c* axis. It is interesting that both TmFeO_3_ single crystal and TbFeO_3_ single crystal exhibit a strong magnetocrystalline anisotropy between *ab* plane and *c* axis, and almost magnetic isotropy in *ab* plane.

The rotating magnetic entropy change 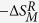 can be obtained by rotating the crystal from *b* to *c* axis and measuring the corresponding isothermal magnetization curves. Figure 6(a,b) indicate the representative isothermal magnetization curves at different angles for temperatures of 8 K and 10 K for TbFeO_3 _single crystal and of 16 K and 18 K for TmFeO_3_ single crystal, respectively. Taking *b* axis as the starting angle, we can get the rotating magnetic entropy change *−*Δ*S*_*M*_^*R*^ as a function of angle by using Eq. (1). As is shown in Fig. 7(a,b), the largest values of −Δ*S*_*M*_^*R*^ = 17.42 J/kg K can be achieved at temperature of 9 K for TbFeO_3 _and *−*Δ*S*_*M*_^*R*^ = −9.01 J/kg K can be achieved at temperature of 17 K for TmFeO_3_ both under a magnetic field of 50 kOe from *b* to *c* axis. Since *R*FeO_3_ (*R* = Tb, Tm) single crystals exhibit almost magnetic isotropy in *ab* plane and a strong magnetocrystalline anisotropy between *ab* plane and *c* axis, Fig. 7(c–d) display the “expected” magnetic entropy change *−*Δ*S*_*M*_^*R*^. As proposed by Kuz’min and Tishin^24^, the large and reversible anisotropic magnetic entropy change with broad temperature span suggests that a promising candidate for new type magnetic refrigeration can be achieved by simply rotating the RFeO_3_ (R = Tb, or Tm) single crystals or magnet.

In conclusion, we investigated the MCE of RFeO_3_ single crystals among *a, b* and *c* axis. The large MCE with broad temperature span and little hysteresis loss is ideal for the application of magnetic refrigeration operated in a wide temperature window. The detailed analysis of magnetization data shows that both TbFeO_3_ single crystal and TmFeO_3_ single crystal exhibit a strong magnetocrystalline anisotropy between *ab* plane and *c* axis and almost magnetic isotropy in *ab* plane. Owing to the difference in charge distribution of 4*f*-elctrons between Tb^3+^ and Tm^3+^ ions, the rotating field entropy with different signs, *−*Δ*S*_*M*_^*R*^ = 17.42 J/kg K, and *−*Δ*S*_*M*_^*R*^ = −9.01 J/kg K are achieved at 9 K and 17 K for TbFeO_3_ and TmFeO_3_ single crystals from *b* axis to *c* axis, respectively. This discovery not only gives us a deeper insight into the understanding of the MCE anisotropy in spin canting anti-ferromagnetic single crystal, but also opens a new arena for rotary magnetic refrigerator by rotating its magnetization vector.

## Methods

TbFeO_3_, TmFeO_3_ and YFeO_3_ ceramic were prepared with the starting material Tb_4_O_7_ (>99.9%), Tm_2_O_3_(>99.9%), Y_2_O_3_(>99.9%) and Fe_2_O_3_(>99.9%) with the ratio of stoichiometric. Then, they were pressed into pellets and sintered in air atmosphere for 48 hours using the solid state reaction method at 1250 °C, 1300 °C and 1300 °C. X-ray diffraction (XRD) patterns showed the prepared samples were single-phase with *P*bnm crystallographic symmetry. The ceramics were compressed into rods under the hydrostatic pressure and sintered at 1400 °C for 48 hours. TbFeO_3_, TmFeO_3_ and YFeO_3_ single crystals were grown with four ellipsoidal mirrors (Crystal Systems Inc, FZ-T-10000-H-VI-VP) by the floating zone method. X-ray diffraction (XRD) patterns were collected by Rigaku D/MAX 2400 x-ray diffractometer with Cu-Kα radiation (λ = 1.5406Å). Back-reflection Laue x-ray diffraction measurements were carried out to determine the crystallographic direction. Magnetization measurements were performed on commercial superconducting quantum interference device (SQUID) magnetometer (Quantum design MPMS-XL).

## Additional Information

**How to cite this article**: Ke, Y.-J. *et al.* Anisotropic magnetic entropy change in *R*FeO_3_ single crystals(R=Tb, Tm, or Y). *Sci. Rep.* **6**, 19775; doi: 10.1038/srep19775 (2016).

## Figures and Tables

**Figure 1:**
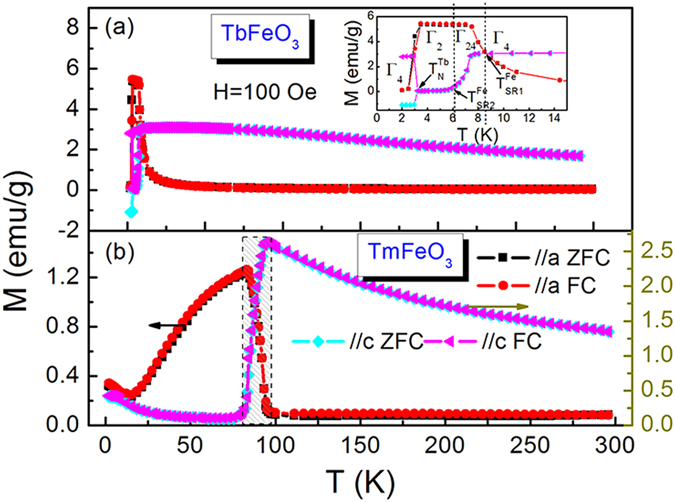
Zero-field-cooled (ZFC) and field-cooled (FC) thermal magnetization curves. (**a**) of TbFeO_3_ along *a* and *c* axis; and (**b**) of TmFeO_3_ along *a* and *c* axis from 2 K to 300 K under a magnetic field of 100 Oe. Insets: thermal magnetization curves along *a* and *c* axis of TbFeO_3_.

**Figure 2:**
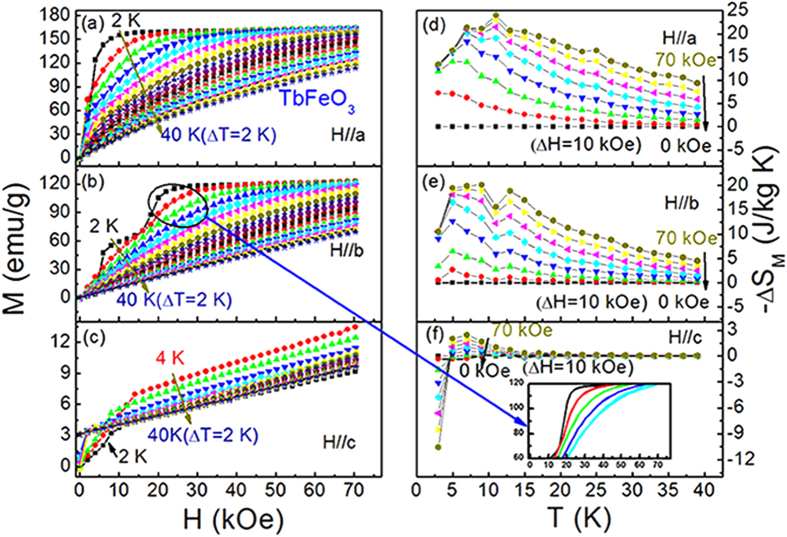
Isothermal magnetization curves and magnetic entropy change of TbFeO_3_ single crystal. (**a**) magnetization curves along *a* axis, (**b**) magnetization curves along *b* axis, (**c**) magnetization curves along *c* axis; (**d**) magnetic entropy change along *a* axis, (**e**)magnetic entropy change along *b* axis, and (**f**) magnetic entropy change along *c* axis.

**Figure 3:**
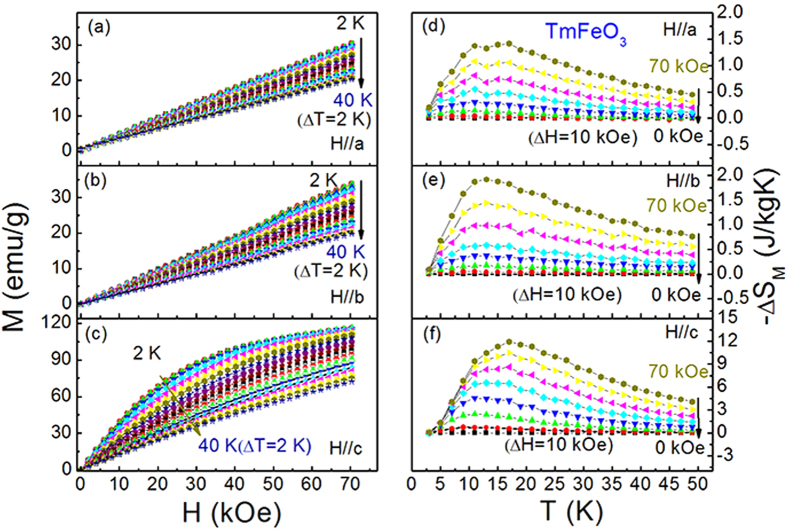
Isothermal magnetization curves and magnetic entropy change of TmFeO_3_ single crystal. (**a**) magnetization curves along *a* axis, (**b**) magnetization curves along *b* axis, (**c**) magnetization curves along *c* axis, (**d**) magnetic entropy change along *a* axis, (e) magnetic entropy changes along *b* axis, and (**f**) magnetic entropy change along *c* axis.

**Figure 4:**
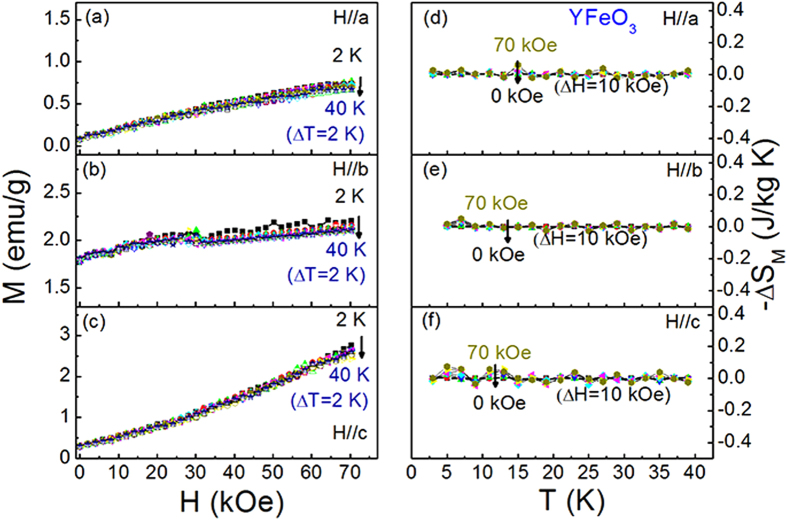
Isothermal magnetization curves and magnetic entropy change of YFeO_3_ single crystal. (**a**) magnetization curves along *a* axis, (**b**) magnetization curves along *b* axis, (**c**) magnetization curves along *c* axis, (**d**) magnetic entropy change along *a* axis, (**e**) magnetic entropy change along *b* axis, and (**f**) magnetic entropy change along *c* axis.

**Figure 5:**
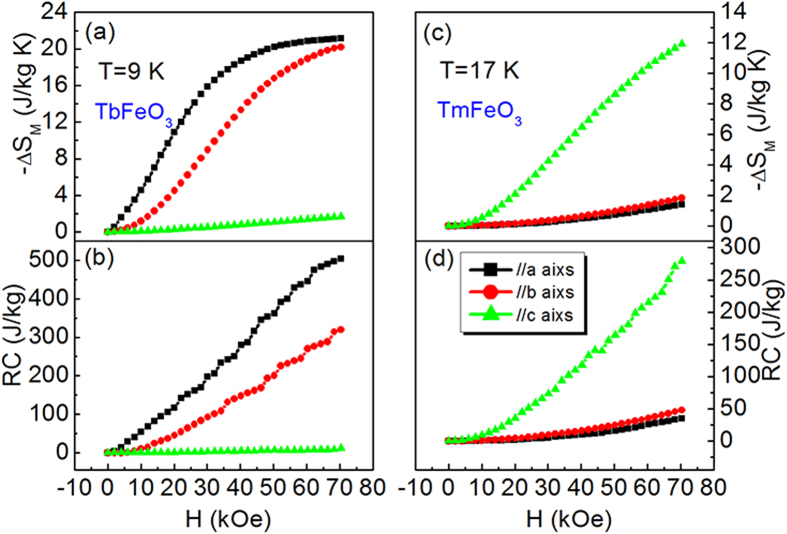
Calculated*−*Δ*S*_*M*_ and refrigeration capacity (*RC*). (**a**) field dependence of magnetic entropy changes of TbFeO_3_ along *a, b* and *c* axis at 9 K, and (**b**) refrigeration capacity; (**c**) field dependence of magnetic entropy change of TmFeO_3_ along *a, b* and *c* axis at 17 K, and (**d**) refrigeration capacity.

**Figure 6:**
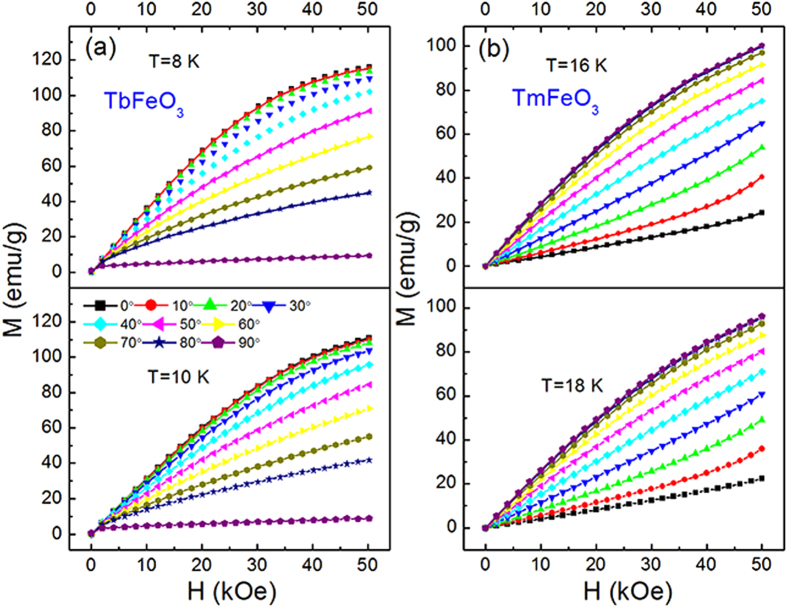
Representative isothermal magnetization curves at different angles in bc plane. (**a**) of TbFeO_3_ single crystal at 8 K and 10 K; (**b**) of TmFeO_3_ single crystal at 16 K and 18 K. 0 and 90 correspond to the *b* and *c* directions, respectively.

**Figure 7:**
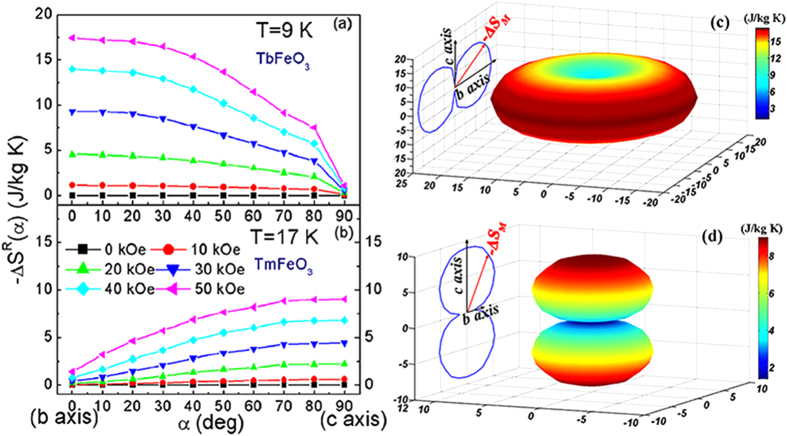
Rotating field entropy changes *−*Δ*S*^*R*^*(α)* from *b* axis to *c* axis vs magnetic field. (**a**) of TbFeO_3_ single crystal at 9 K; (**b**) of TmFeO_3_ single crystal at 17 K; (**c**) “expected” anisotropy of magnetic entropy change of TbFeO_3_ single crystal; and (**d**) “expected” anisotropy of magnetic entropy change of TmFeO_3_ single crystal.
